# Expression Profiling of *Cucumis sativus* in Response to Infection by *Pseudoperonospora cubensis*

**DOI:** 10.1371/journal.pone.0034954

**Published:** 2012-04-24

**Authors:** Bishwo N. Adhikari, Elizabeth A. Savory, Brieanne Vaillancourt, Kevin L. Childs, John P. Hamilton, Brad Day, C. Robin Buell

**Affiliations:** 1 Department of Plant Biology, Michigan State University, East Lansing, Michigan, United States of America; 2 Department of Plant Pathology, Michigan State University, East Lansing, Michigan, United States of America; Technical University of Denmark, Denmark

## Abstract

The oomycete pathogen, *Pseudoperonospora cubensis*, is the causal agent of downy mildew on cucurbits, and at present, no effective resistance to this pathogen is available in cultivated cucumber (*Cucumis sativus*). To better understand the host response to a virulent pathogen, we performed expression profiling throughout a time course of a compatible interaction using whole transcriptome sequencing. As described herein, we were able to detect the expression of 15,286 cucumber genes, of which 14,476 were expressed throughout the infection process from 1 day post-inoculation (dpi) to 8 dpi. A large number of genes, 1,612 to 3,286, were differentially expressed in pair-wise comparisons between time points. We observed the rapid induction of key defense related genes, including catalases, chitinases, lipoxygenases, peroxidases, and protease inhibitors within 1 dpi, suggesting detection of the pathogen by the host. Co-expression network analyses revealed transcriptional networks with distinct patterns of expression including down-regulation at 2 dpi of known defense response genes suggesting coordinated suppression of host responses by the pathogen. Comparative analyses of cucumber gene expression patterns with that of orthologous *Arabidopsis thaliana* genes following challenge with *Hyaloperonospora arabidopsidis* revealed correlated expression patterns of single copy orthologs suggesting that these two dicot hosts have similar transcriptional responses to related pathogens. In total, the work described herein presents an in-depth analysis of the interplay between host susceptibility and pathogen virulence in an agriculturally important pathosystem.

## Introduction

Cucumber (*Cucumis sativus* L.) is an economically important vegetable crop cultivated in over 80 countries, with more than 66 million tons produced annually for both fresh use and processing (http://faostats.fao.org). Cucumber has been utilized extensively as a model system to study sex determination [1], vascular biology [2], and induced defense responses [3], [4]. Despite its extensive use as a model system in research, as well as its obvious economic importance, genetic and genomic resources remain limited. In recent years, the publication of both a genetic map [5] and genome sequences [6], [7] of cucumber, as well as generation of large-scale expression data sets [8], [9], have provided the first comprehensive resources for genetic and genomic based inquiries into cucumber biology. Cucumber has limited genetic diversity, few wild relatives, and only 7 pairs of chromosomes (2n = 14), whereas other *Cucumis* spp., such as melon (*Cucumis melo*), have 12 chromosomes, making interspecific breeding difficult, if not impossible. As such, advances in breeding for important agronomic traits such as increased yield, fruit quality, and disease resistance are slow.

Cucumber production is hindered by diseases caused by bacterial (e.g., *Pseudomonas syringae* pv. *lachrymans*), viral (e.g., *Cucumber mosaic virus*), fungal (e.g., *Sphaerotheca fulginea* and *Erysiphe cichoracearum*), and oomycete (e.g., *Phytophthora capsici* and *Pseudoperonospora cubensis*) pathogens [10], [11]. Of these, the most destructive is *Ps. cubensis*, the causal agent of cucurbit downy mildew, which threatens cucumber production in nearly 80 countries and causes severe economic losses [11], [12], [13]. *Ps. cubensis* is an obligate, biotrophic oomycete pathogen with a host range limited to the *Cucurbitaceae* [11]. In recent years, a foundation has been established to support advances in this area, including studies on epidemiology [14], host specificity [15], [16], [17], [18], pathogenic variation [19], [20], [21], and more recently, generation of a draft genome sequence of *Ps. cubensis* [22], [23].

The molecular and biochemical mechanisms associated with host resistance have been investigated to a limited extent in cucumber and other cucurbits. In large part, signaling of resistance is primarily associated with systemic acquired resistance (SAR) [24], for which cucumber has historically been a model system [25], [26]. In addition to SAR, structural modifications (e.g., callose deposition), as well as the induction of defense-related genes (e.g., peroxidases, chitinases, and glucanases) are often associated with the onset of resistance signaling following pathogen infection. Moreover, like other well-characterized plant-pathogen systems, the presence of nucleotide-binding site (NBS) containing genes encoding protein products that recognize cognate pathogen effector proteins or their perturbations [27] have been postulated to play a role in disease resistance in cucumber. To this end, analysis of the cucumber genome sequence has identified 61 NBS resistance genes, considerably less than have been identified in other plant genomes, such as Arabidopsis (200) or rice (600) [6]. However, of the 15 genes known to control disease resistance in cucumber, none have been cloned, nor have they been associated through linkage maps with the 61 predicted NBS resistance genes [28]. It is hypothesized that cucumber has an expanded lipoxygenase pathway which may provide an additional mechanism(s) to facilitate responses to biotic stress [6].

While genetically conferred host resistance is the ideal means of disease control in crop species, it has become ineffective in controlling cucurbit downy mildew, particularly in the U.S., where introduction of a new, more virulent pathotype of *Ps. cubensis* is responsible for economic losses in recent years [12], [29]. To this end, control methods for cucurbit downy mildew in both Europe and the U.S. require the use of fungicides, coupled with a single host resistance locus, the recessive *dm1* gene, which has been incorporated into most commercial cucumber germplasm [11]. However, the identification of the *dm1* locus, as well as its functional role in resistance signaling remains undefined. In addition to widespread incorporation of *dm1*, breeding of *Ps. cubensis* resistance has focused mainly on genes from melon [30], as limited diversity for resistance is available in cucumber or its wild relative, *Cucumis hardwickii*. Large-scale screening trials to identify tolerant germplasm are in progress, but have yet to identify a source of complete resistance to *Ps. cubensis* [16], [31]. As such, new resources must be explored to support development of improved cultivars and identify new sources of resistance for breeding programs.

Next generation sequencing of the transcriptome (mRNA-Seq) permits deep, robust assessments of transcript abundances and transcript structure [32]. When gene expression profiling is applied to host-pathogen interactions of economically important crops, insights into the mechanisms these pathogens use to suppress and subvert the host defense response can be made [33], [34], [35]. In the current study, we performed expression profiling, using the susceptible cucumber cultivar ‘Vlaspik’, over a time course of infection with the downy mildew pathogen *Ps. cubensis* to identify genes, pathways, and systems that are altered during a compatible interaction. Using this technology, deep profiling of both the host and pathogen transcriptome (see accompanying manuscript [36]) was achieved, providing the first in-depth analysis of this important plant-pathogen interaction. In this study, we cataloged the expression of 14,476 genes from cucumber through an 8-day time course of infection with a virulent *Ps. cubensis* isolate. In total, this work identified major changes in gene expression in cucumber at 1 day post-inoculation (dpi) continuing through 8 dpi, with up to 3,286 genes differentially expressed between time points. Comparative analyses revealed correlated gene expression patterns in cucumber and *Arabidopsis thaliana* leaves infected with downy mildew, suggesting orthologous host responses in these two dicotyledonous hosts. Through co-expression network analyses, modules of temporal-specific transcriptional networks were constructed that provide a framework to connect transcription factors with defense response genes.

## Results and Discussion

### Response of C. sativus leaves to pathogen infection

**Figure 1:**
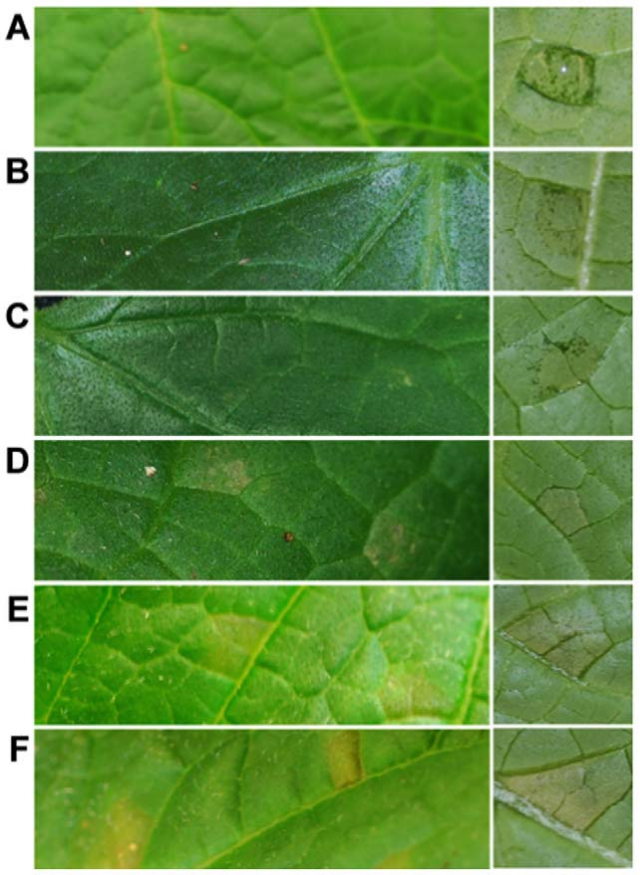
Symptoms of *Pseudoperonospora cubensis* infection on susceptible *Cucumis sativus* cv. ‘Vlaspik’. Images were collected of the adaxial (left column) and abaxial (right column) leaf surfaces at 1, 2, 3, 4, 6, and 8 days post-inoculation (dpi). **A**., 1 dpi, **B**., 2 dpi, **C**., 3 dpi, **D**., 4 dpi, **E**., 6 dpi, **F**., 8 dpi.

To correlate gene expression and host responses with observable disease symptoms and pathogen invasion, the progression of infection in susceptible *C. sativus* cv. ‘Vlaspik’ was monitored at 1, 2, 3, 4, 6, and 8 dpi. As shown in Figure 1, the first visible symptoms of *Ps. cubensis* infection were apparent at 1 dpi, in the form of water soaking on the abaxial leaf surface at the inoculation site (Figure 1A). These symptoms correspond to zoospore encystment and initial penetration through the stomata into the host [36]. In similar pathosystems, systems such as *Hyaloperonospora arabidopsidis*, the causal agent of downy mildew on *A. thaliana*, analogous processes occur in the early stages of infection. One exception is that *H. arabidopsidis* penetrates between anticlinal walls of epidermal cells rather than utilizing natural openings like stomata [37]. While no symptoms are visible on the upper leaf surface in cucumber, water soaking on the lower leaf surface can be seen as early as 1 dpi, and remains present through 4 dpi, during which time hyphal growth through the mesophyll of the host tissue occurs and haustoria formation begins [36]. Yellow angular lesions bound by leaf veins characteristic of cucurbit downy mildew were first visible on the upper leaf surface at 4 dpi (Figure 1D), and became more chlorotic and slightly necrotic at the centers by 8 dpi. These symptoms are associated with extensive growth of hyphae through the plant mesophyll [36].

### Gene expression profiling in *C. sativus*

**Figure 2:**
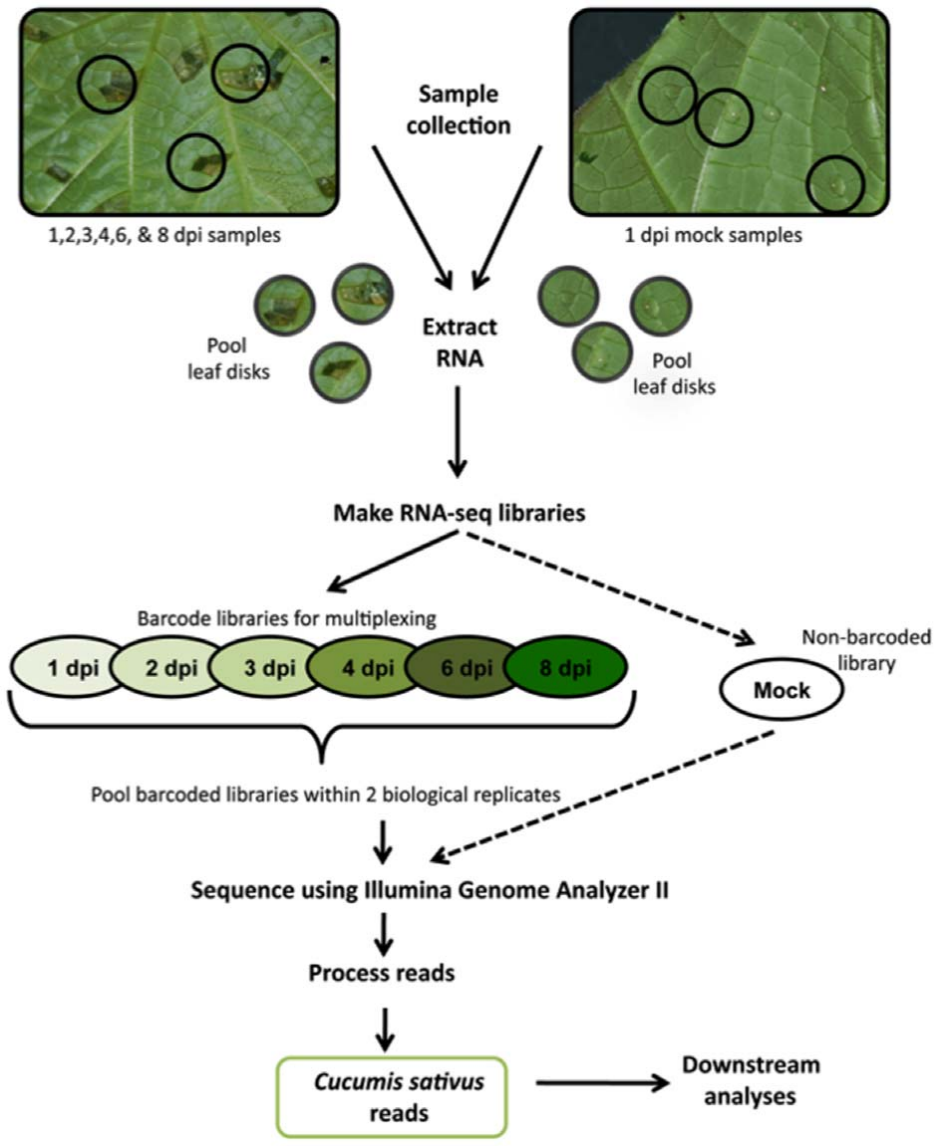
Experimental design and sample collection. Cucumber cv. ‘Vlaspik’ leaves were inoculated on the abaxial leaf surface with 10–30 10 μl droplets of a 1×10^5^ sporangia/ml solution. Samples were collected at 1, 2, 3, 4, 6, and 8 days post-inoculation (dpi) using a #3 cork borer to isolate tissue immediately around the infection point (black circles). Samples from cucumber leaves mock-inoculated with 10 μl droplets of dH_2_O were collected in the same manner at 1 dpi. Leaf disks from each time point were pooled for RNA extraction. mRNA-Seq libraries were made for each time point from two separate biological replicates. Within a biological replicate, libraries were barcoded and sequenced in multiple lanes with the exception of the mock-inoculated library, which was not barcoded.

**Figure 3:**
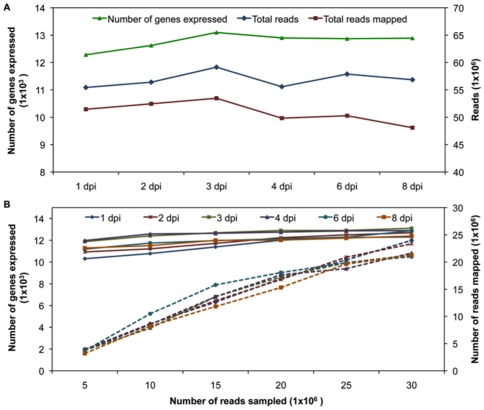
Comparison of total mRNA-Seq reads, reads mapped and number of genes expressed. **A**. Total number of reads, total reads mapped, and number of genes expressed as determined from pooling of both biological replicates are shown. Reads were mapped to the *C. sativus* genome [6] using Bowtie version 0.12.5 [57] and TopHat version 1.2.0 [56]. Fragments per kilobase pair of exon model per million fragments mapped (FPKM) values for the annotated *C. sativus* genes were calculated using Cufflinks version 0.9.3 [58]. Genes were considered expressed if the FPKM value and 95% confidence interval lower boundary FPKM value was greater than 0.001 and zero, respectively. **B**. Effect of sampling depth on detection of expressed genes. For all time points 5, 10, 15, 20, 25, and 30 million reads were randomly selected from the total pool of reads. Read mapping and expression abundance estimates were performed as described above. Solid lines indicate number of genes expressed and the dashed lines indicate number of reads mapped at different time points. dpi, days post-inoculation.

We performed mRNA-Seq profiling of *C. sativus* leaves following infection with *Ps. cubensis* over an 8-day period. Leaf disc samples were collected using a cork borer to maximize the amount of infected tissue in each sample (Figure 2, black circles), pooled within a given time point, and RNA was isolated. Two biological replicates of each time point were sequenced, yielding 55 to 59 million reads from both replicates at each time point. Additionally, a mock-inoculated *C. sativus* sample was collected and sequenced, yielding 5.8 million reads. The number of reads that mapped to the *C. sativus* genome ranged from 48 to 53 million (Figure 3A, 84–93% of the total reads) per time point while the number of genes expressed at different time points ranged from 12,257 to 13,048 (Figure 3A).

The libraries were constructed from inoculated leaves and therefore, the reads represent transcripts from the host (*C. sativus*) and the pathogen (*Ps. cubensis*). At the early time points, nearly all of the reads obtained were of host origin, which is consistent with our microscopy analysis revealing limited pathogen biomass. However, as we are surveying a susceptible interaction, pathogen biomass increases throughout the time course and consequentially, pathogen transcripts increase in the total read pool in the later time points [36]. However, even with the increased percentage of pathogen reads in the later time points, we have saturated sampling of the *C. sativus* transcriptome with our deep read coverage of the libraries. Randomly selected subsets of reads, 5 to 30 million, from the total read pool were used to evaluate the effect of sampling depth on gene expression detection. The simulation demonstrates a positive relationship between sampling depth and numbers of expressed genes at lower sequencing depths (5 to 20 million reads) (Figure 3B). The number of expressed genes, however, begins to plateau at approximately 20 million reads, corresponding to the minimum sampling depth of all libraries in this study. To study the repeatability of two biological replicates, pair-wise scatter plots of gene expression values were generated. With correlation coefficients (*R^2^*) ranging from 0.97 to 0.98 for biological replicates of each time point, nearly all genes fell along the diagonal of plots, indicating no major variation and providing evidence for high reproducibility of biological replicates (Figure S1).

### Host transcriptional changes in response to infection

Over the infection period, a total of 14,476 *C. sativus* genes were expressed (Table S1), with 10,350 genes common to all time points. For all data points analyzed, the minimum fragments per kilobase exon model per million mapped reads (FPKM) value was zero, yet the maximum FPKM values ranged from 8,869 at 1 dpi to 34,017 at 8 dpi (Table S1). Interestingly, the highest up-regulated gene at 1 dpi was a putative galactinol synthase (Csa6M000080.1), which has been shown to be up-regulated in *Cucumis melo* (melon) in response to abiotic stress [38] as well as in an inbred *C. sativus* line ‘IL57’ with a high level of resistance to downy mildew [39]. The expression patterns of the top 20 highly expressed genes showed expression of genes involved in defense responses including catalases, chitinases, lipoxygenases, peroxidases, and protease inhibitors, beginning at 1 dpi and extending through 8 dpi (Table S2). The detection of defense-related genes within 1 dpi suggests that there is an active response by *C. sativus* to early infection stages of *Ps. cubensis*, including zoospore encystment, appressorium formation, and penetration via stomata [36]. Additionally, no pathogen defense-related genes are present within the top 20 highly expressed genes in the mock-inoculated samples, which mainly consists of housekeeping genes (Table S2).

**Figure 4:**
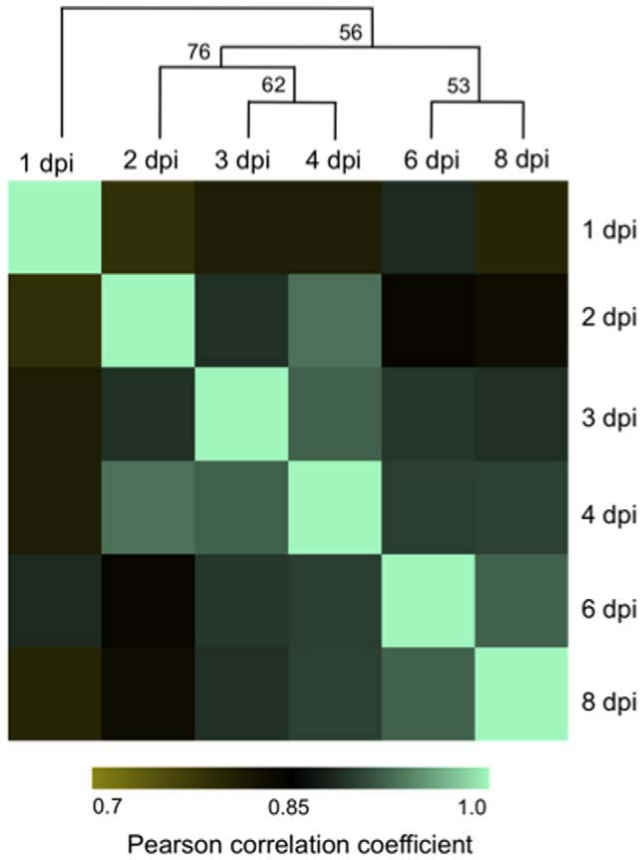
Correlation matrix of *Cucumis sativus* expression profiles during infection by *Pseudoperonospora cubensis*. Tissue samples were collected from *C. sativus* at different time points of infection with *Ps. cubensis*. Normalized transcript abundances for 14,476 genes were calculated as fragments per kilobase pair of exon model per million fragments mapped (FPKM) with Cufflinks version 0.9.3 [58]. Pearson correlation coefficient of log2 FPKM values were calculated for all pair-wise comparisons using R (http://cran.r-project.org/). Hierarchical clustering was performed using Pearson correlation distance metric and average linkage with the Multiple Experiment Viewer Software version 4.5 [60]. The bootstrap support values shown on tree nodes were obtained from 1000 bootstrap replicates. dpi, days post-inoculation.

Correlation and cluster analyses were used to identify similarities in transcriptome profiles among the sampled time points. Pearson Correlation Coefficient (PCC) values for time point comparisons ranged from 0.78 to 0.93, with tight clustering readily apparent, revealing patterns that highlight the extent of transcriptional diversity underlying early (1 dpi), intermediate (2, 3, and 4 dpi), and advanced (6 and 8 dpi) stages of disease progression and the corresponding responses in host gene expression (Figure 1). As described above, *C. sativus* defense-related genes are expressed within 1 dpi of *Ps. cubensis* inoculation, and based on our correlation analysis (Figure 4), these likely represent a distinct cluster of genes responding specifically to initial recognition of sporangia, germination of zoospores, and zoospore encystment in stomata. Genes expressed at 2–4 dpi also cluster more with each other than to 1 dpi or to later time points, which also reflects the similar stages of *Ps. cubensis* infection apparent at this stage, indicating that these genes may be involved in host responses to hyphal penetration, hyphal growth and haustorium formation. The clustering of gene expression at later time points (6 and 8 dpi) likely corresponds to similar symptoms observed (Figure 1) as well as plant responses to extensive *Ps. cubensis* hyphal growth that is occurring at those time points [36].

### Conserved host responses in *C. sativus* and *A. thaliana* in compatible interactions as measured through expression profiling

**Figure 5:**
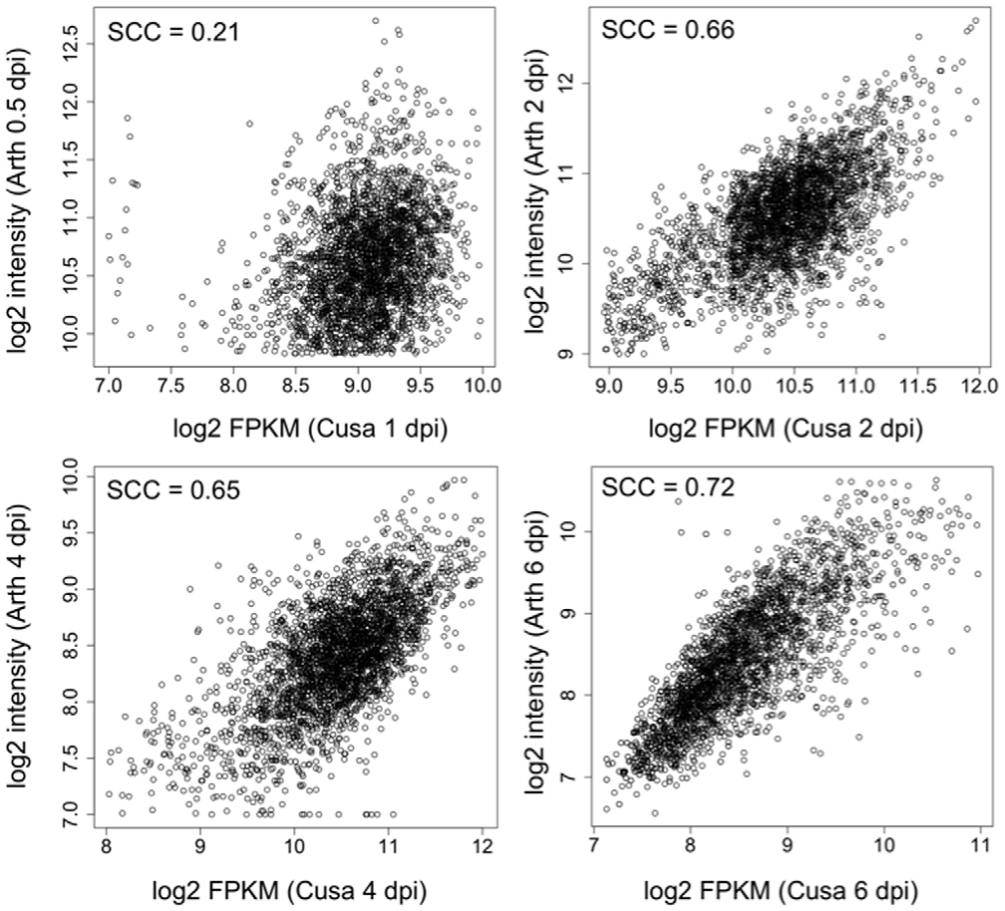
Comparison of orthologous gene expression in *Cucumis sativus* and *Arabidopsis thaliana* in a compatible interaction. Microarray expression profiles were obtained from time-course analyses of genes expressed in *A. thaliana* (Arth) during infection by *H. arabidopsidis* [41]. Single copy orthologous genes between *C. sativus* (Cusa) and *A. thaliana* were identified using OrthoMCL [63]. Log2 transformed expression values of single copy orthologous genes expressed in the *C. sativus* mRNA-Seq dataset (log2 fragments per kilobase pair of exon model per million fragments mapped [FPKM]) and *A. thaliana* microarray dataset (log2 intensity) are shown as scatter plots. SCC, Spearman correlation coefficient; dpi, days post-inoculation.

Host responses to pathogen challenge have been well documented in the model species *A. thaliana* [40], including those to the downy mildew pathogen *H. arabidopsidis* [34], [37]. To identify genes induced in a compatible interaction with a downy mildew pathogen common to both *C. sativus* and *A. thaliana*, we identified single copy orthologous genes in both plant genomes and analyzed their expression patterns. A total of 7,396 clusters of single copy genes from both species were identified by clustering 23,248 and 27,416 representative protein coding genes from *C. sativus* and *A. thaliana*, respectively. Data from a previous microarray-based expression profiling [41] experiment of a compatible *A. thaliana-H. arabidopsidis* interaction was compared with our mRNA-Seq-based expression data. The *H. arabidopsidis* infection time points were 0, 0.5, 2, 4, and 6 dpi, similar to the 1–8 dpi time points assayed in this study. Spearman rank correlation coefficients (SCCs) of log2 expression values were calculated between the single copy orthologs at all time points in the two datasets; between 2,136 and 3,446 gene pairs were included in the pair-wise comparisons. Among the six comparisons between similar time points, the SCC values ranged from 0.10 to 0.72 (Table S3). The correlations between early time points were the lowest between the two interactions, possibly due to the differences in penetration strategies between the two pathogens. The highest correlations were observed between 6 dpi (SCC = 0.72) followed by 2 dpi (SCC = 0.66) for both host-pathogen interactions (Figure 5). Overall, correlation coefficients between analogous time points (0.65 to 0.72) were greater than comparisons between dissimilar time points (0.10 to 0.33) indicating similar expression patterns for single copy orthologous genes in *C. sativus* and *A. thaliana*.

### Differential gene expression throughout the infection process

Differences in gene expression patterns between time points can provide insight into the host response following pathogen perception and subsequent infection; thus, the mechanism(s) through which pathogenicity occurs may be inferred [42], [43], [44], [45]. For example, a recent publication by Gaulin et al. [46] used a comparative approach analyzing the transcriptomes of *Aphanomyces euteiches* and *Phytophthora* spp. to identify novel pathogenicity factors and expanded repertoires for virulence. Through comparison of gene expression patterns across time points, we identified genes differentially expressed between all time points and the control sample, 1 dpi mock inoculation; between 1,170 and 3,286 genes were differentially expressed in pair-wise comparison between the mock inoculated and/or the inoculated time points (Table 1, Table S4). In general, 12 to 31% of the genes tested under different conditions were differentially expressed in the pair-wise comparisons. In infected samples, comparisons of the three early to intermediate time points (i.e., 1, 2, and 3 dpi) showed a higher number of differentially expressed genes (2,214 to 3,286) than those between three intermediate to later (4, 6 and, 8 dpi) time points (1,612 to 2,074), suggesting more correlated gene expression in later stages of infection.

**Table 1:** Number of genes differentially expressed between different time points.

	1 dpi¥	2 dpi	3 dpi	4 dpi	6 dpi	8 dpi
Mock-Control	1,170 (12%)§	1,906(19%)	2,596 (26%)	2,509 (25%)	1,713 (17%)	2,556 (26%)
1 dpi		3,286 (31%)	2,948 (30%)	2,849 (27%)	2,010 (20%)	3,014 (30%)
2 dpi			2,214 (23%)	1,736 (18%)	2,718 (27%)	3,006 (29%)
3 dpi				1,590 (16%)	1,840 (18%)	2,218 (23%)
4 dpi					1,842 (18%)	2,074 (21%)
6 dpi						1,612 (16%)

Differential expression analysis was conducted using the cuffdiff program in Cufflinks version 0.9.3 [58], *Cucumis sativus* v2 annotation, and false discovery rate of 0.01.

¥ dpi, days post-inoculation.

§ Numbers in parenthesis indicate the percent of significantly different tests out of the total number of tests that could be performed for each pair-wise comparison.

### Gene co-expression pattern analyses

**Figure 6:**
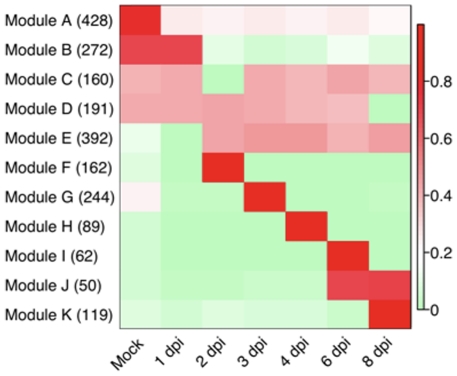
Heat map of eigengenes representing each gene module. The mock control and post-infection time points are represented in columns and the eigengenes for each of the 11 identified coexpression modules [49] are presented in rows. The numbers of genes in each module are given in parentheses. The eigengene values, which range from 0 to 1, are a measure of centrality and indicate the relative expression levels of all genes in the module (see Materials and Methods). dpi, days post-inoculation.

**Figure 7:**
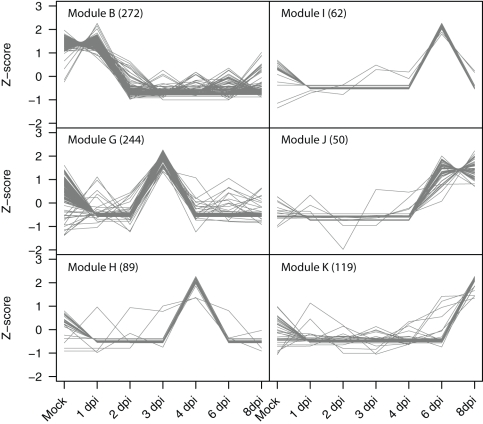
Trend plots of the normalized gene expression values from six identified gene co-expression modules. Modules consisting of genes down-regulated at 2 dpi (Module B), genes up-regulated at a single time point (Modules G, H, I, K), and genes up-regulated at two time-points (Module J) are shown. The number of genes in each module is shown in parentheses.

Using Weighted Gene Correlation Network Analysis (WGCNA) [47], we identified sets of highly correlated genes and constructed modules where all members are more highly correlated with each other than they are to genes outside the module [48]. Out of 15,286 genes expressed in the mock control or throughout the infection time course, 4,410 genes passed the Coefficient of Variance (CV) filter (0.4) and were retained for downstream analyses. Of the 4,410 genes, a total of 2,169 were assigned to 11 gene modules that contained between 50 and 428 genes (Table S5); 2,241 genes were not assigned to any module. To visualize the relationship of the modules to each other with respect to the progression of infection, eigengenes for each module were calculated and displayed in a heat map [49]. As shown in Figure 6, some modules are representative of genes with correlated co-expression at primarily a single or two time points (Modules F, G, H, I, J, and K) whereas other modules represent genes that share broader co-expression patterns across multiple time points (Modules A, B, C, D, E). Examination of trend plots for the modules (Figure 7, Figure S2) reveals the direction and magnitude of gene expression patterns. Genes within Module B are expressed in the mock control and at 1 dpi, but are coordinately down-regulated at 2 dpi, remaining less abundant through 8 dpi (Figure 7). This set of 272 genes includes a large suite of genes implicated in resistance including six lipoxygenase genes, four cationic peroxidases, two cinnamate 4-hydroxylases, an anthocyanidin 3-glucosyltransferase, an anthocyanin 5-aromatic acyltransferase, a cysteine protease, and the elicitor-inducible protein EIG-J7. All of these genes have been implicated in defense responses in other plants [50], [51], [52], [53], [54], [55], and in total, their coordinate down-regulation at 2 dpi is suggestive of an active mechanism by the pathogen to alter host defense responses. For example, the lipoxygenase pathway has been hypothesized to be involved in defense responses in cucumber, due to its expansion within the genome [6], and our data presented herein showing a rapid down-regulation of these transcripts during infection supports this hypothesis. In addition, cinnamate 4-hydroxylase, one of the primary enzymes in the phenylpropanoid pathway responsible for the conversion of cinnamic acid to *p*-coumaric acid, was shown to be rapidly induced in *C. sativus* in response to abiotic stress [52]. In this context, it is reasonable to hypothesize that the down-regulation of its expression (observed in the present study), as well as that of other genes within the phenylpropanoid pathway, are suggestive of an active virulence mechanism to abrogate host responses associated with stress-induced signaling. In addition to the direct correlation among defense gene expression noted above, this module also includes the coordinated expression of 33 transcription factors that may also have critical roles in regulating genes responsible for the induction of defense signaling in the host. A total of 33 genes of no known function are also in this module, and further examination of their roles in defense responses may provide new insight into critical host genes modulated by virulent pathogens.

Modules F, G, H, I, J, and K all have discrete time points where genes are up-regulated compared to the other sampled time points. As shown in Figure S2, genes in Module F (162 genes total) have a peak of expression solely at 2 dpi. This module contains 21 transcription factors which could be key to regulating genes from Module G that have peak expression at 3 dpi (Figure 7). Likewise, Module G (244 genes total) has 21 transcription factors that may regulate genes within Module H (89 genes total) that are coordinately up-regulated at 4 dpi (Figure 7). Within all of these modules (F, G, H, I, J, and K, 726 genes total) there are 199 genes with no known function and placement of these genes in transcriptional networks provides a new functional annotation and contextual information on their function.

In summary, the work described herein represents the first genome-scale analysis of the cucumber-downy mildew interaction in which we catalog gene expression throughout an 8 day infection period and identify differentially expressed genes that could be correlated with pathogen growth and development *in planta*. With expression profiles for nearly 15,000 genes during a compatible interaction, we have new insight into molecular events at the host-pathogen interface including a suite of defense response-related genes that are down-regulated early upon infection and transcriptional networks that respond in a temporal manner throughout the infection cycle. Most intriguingly, these networks include transcription factors and genes of no known function, which may have a role in the host-pathogen interaction.

## Materials and Methods

### Plant materials, inoculum and pathogen infection

*C. sativus* cv. ‘Vlaspik’ was grown in growth chambers maintained at 22°C with a 12 h light/dark photoperiod. *Ps. cubensis* MSU-1 was maintained as previously described [22]. The first fully expanded leaf of four-week-old cucumber plants was inoculated on the abaxial surface with 20–30 10 μl droplets of a 1×10^5^ sporangia/ml solution. After inoculation, plants were kept at 100% relative humidity for 24 hours in the dark. Plants were returned to growth chambers for disease progression.

### Sampling and experimental design

Samples from two biological replicates were collected at 1, 2, 3, 4, 6, and 8 dpi at the site of inoculation using a #3 cork borer. Additionally, a mock-inoculated sample (i.e.,10 μl dH_2_O), which was inoculated as described above and kept at 100% relative humidity for 24 hours in the dark was collected at 1 dpi. Samples were frozen immediately in liquid nitrogen and stored at −80°C until use.

### Library preparation and sequencing

Total RNA was isolated from infected leaf discs using the RNeasy Mini Kit (Qiagen, Germantown, MD) and treated with DNase (Promega, Madison, WI) per the manufacturer's instructions. RNA concentration and quality was determined using the Bioanalyzer 2100 (Agilent Technologies, Santa Clara, CA). The mRNA-Seq sample preparation was done using the Illumina mRNA-Seq kit (Illumina, San Diego CA) according to the manufacturer's protocol. Parallel sequencing was performed using an Illumina Genome Analyzer II (Illumina, Inc., San Diego, CA) at the Research Technology Support Facility (RTSF) at Michigan State University. Each library within a biological experiment was barcoded, six different barcodes for 6 time-points, pooled and run on multiple lanes. Two biological replicates of each time-point were sequenced multiple times and single-end reads between 35 and 42 bp were generated. Reads from both biological replicates were pooled for determining expression abundances. The mock-inoculated sample library was made as described above, but not barcoded and run in a single lane.

### Processing of mRNA-Seq data

mRNA-Seq reads obtained from Illumina Pipeline version 1.3 were quality evaluated on the Illumina purity filter, percent low quality reads, and distribution of phred-like scores at each cycle. Reads were deposited in the National Center for Biotechnology Information Sequence Read Archive under accession number SRP009350. Reads in FASTQ formats were aligned to the *C. sativus* [6] reference genome using TopHat v1.2.0/Bowtie v0.12.5 [56], [57]. A reference annotation of *C. sativus* (version 2) from the Cucurbit Genomics Database (http://www.icugi.org/cgi-bin/ICuGI/misc/download.cgi) was provided in which a representative isoform, the gene model with the longest CDS, was used to estimate expression of the gene; a total of 23,248 gene models from a total of 25,600 gene models were used. All other isoforms were discarded from the annotation set. The minimum and maximum intron length was set to 5 and 50,000 bp, respectively; all other parameters were set to the default values.

Normalized gene expression levels were calculated using Cufflinks v0.9.3 [58] using the quartile normalization option [59] to improve differential expression calculations of lowly expressed genes. The maximum intron length parameter was set to 50,000 bp. All other parameters were used at the default settings. Sampling depth was evaluated on expression estimates by randomly selecting 5, 10, 15, 25, and 30 million reads from the total pool of reads at all time points. Pearson correlation coefficient analyses of log2 FPKM values were performed using R (http://cran.r-project.org/), in which all log2 FPKM values less than zero were set to zero. Only tests significant at *p* = 0.05 are shown. The correlation values were clustered with hierarchical clustering using a Pearson correlation distance metric with average linkage and depicted as a heat map. For each node, bootstrap support values were calculated from 1000 replicates using Multiple Experiment Viewer Software (MeV) v4.5 [60]. To examine biological variation, PCC was calculated for the log2 transformed FPKM values of the genes expressed in both replicates at a particular time point.

### Microarray data acquisition and processing

Comparative analyses of host gene expression responses during a compatible interaction with the model species *A. thaliana* utilized microarray-based gene expression data from an *H. arabidopsidis*-*A. thaliana* time course experiment [41]. The dataset was comprised of *A. thaliana* genes expressed in response to *H. arabidopsis* infection at 0, 0.5, 2, 4, and 6 dpi using the ATH1 Affymetrix platform. The probe intensity values were downloaded from Gene Expression Omnibus (http://www.ncbi.nlm.nih.gov/geo/; GSE22274) [61], normalized using Robust Multichip Analysis method [62]. OrthoMCL [63] was used to identify clusters of orthologs and close paralogs in cucumber and *A. thaliana.* For the mRNA-Seq to microarray comparative analysis only single copy orthologous genes were considered for further analyses. The FPKM and intensity values were log2 transformed, and Spearman rank correlations of the single copy orthologs in both hosts were calculated in R (http://cran.r-project.org/). Only tests significant at *p* = 0.05 are shown.

### Identification of differentially expressed genes

The Cuffdiff program within Cufflinks version 0.9.3 [58] was used to identify differentially expressed genes using pair-wise comparisons of the six time points and the control. The minimum number of alignments at a gene required to test was set to 100. Quartile normalization and a false discovery rate of 0.01 after Benjamini-Hochberg correction for multiple testing were used. Significance in the numbers of differentially expressed genes between time points was tested using the two-sample t-test.

### Functional analyses of differentially expressed genes

Functional annotation for all *C. sativus* genes were generated from BLAST searches of the UniProt databases (Uniref100) [64] and combined with Pfam (version 24.0, [65]) protein families assignment using PfamScan (ftp://ftp.sanger.ac.uk/pub/databases/Pfam/Tools/) *C. sativus* sequences were functionally annotated based on the best possible UniRef sequence match using a minimum E value cutoff of 1E-5. If there was no UniRef sequence match, functional annotations were assigned using Pfam domains. Transcription factors were annotated based on PFAM domain assignment.

### Gene co-expression network analysis

Gene modules of highly correlated genes were identified using the WGCNA method [48] implemented in R (http://cran.r-project.org/). All gene FPKM expression values were log2 transformed and any transformed FPKM value less than 1 was converted to zero. Genes without variation across the mock sample and 6 time points were filtered out using a Coefficient of Variance (CV = σ/μ) cutoff of 0.4. The β and treecut parameters for WGCNA were 13 and 0.9, respectively. All other parameters were used with their default values. Eigengenes for each gene module [49] were calculated and presented as a heat map.

## Supporting Information

Figure S1 **Concordance of expression values in two biological replicates of** ***Cucumis sativus*** **during infection by** ***Pseudoperonospora cubensis*** **.** Reads from different time points were mapped to the *C. sativus* genome using Bowtie version 0.12.5 [57] and TopHat version 1.2.0 [56]. Fragments per kilobase pair of exon model per million fragments mapped (FPKM) values were calculated using Cufflinks version 0.9.3 [58] and the *C. sativus* genome annotations. For each time point, log2 transformed FPKM values of equal number of genes from both replicates are plotted. *R^2^*, correlation coefficient; dpi, days post-inoculation.
(PDF)

Figure S2 **Trend plots for all 11 modules.** All 11 modules generated using WGCNA are shown (Modules A through K). The number of genes in each module is shown in parentheses.
(PDF)

Table S1 **List of** ***Cucumis sativus*** **genes expressed following infection with** ***Pseudoperonospora cubensis*** **.** Gene ID, expression values (fragments per kilobase pair of exon model per million fragments mapped, FPKM) at different time points, and their functional annotation are shown.
(XLS)

Table S2 **List of the top 20 genes highly expressed at different time points and control, their FPKM** (fragments per kilobase pair of exon model per million fragments mapped) **values and putative function as determined by BLASTX searches against UniRef100 (E-value cutoff 1e-5), and Pfam** **protein family and domain search.**
(XLS)

Table S3 **Spearman rank correlations of expression values between single copy orthologous genes in** ***Cucumis sativus*** **and** ***Arabidopsis thaliana*** **following infection with a compatible oomycete pathogen.**
(XLS)

Table S4 **List of genes differentially expressed at different time points along with their expression values (FPKM) and functional annotation.** Differential expression analysis was conducted using the cuffdiff program in Cufflinks version 0.9.3 [58]. FPKM, fragments per kilobase pair of exon model per million fragments mapped.
(XLS)

Table S5 **List of modules (Module A through K) with their corresponding module name, gene ID, and putative function as determined by BLASTX searches against UniRef100 (E-value cutoff 1e-5) and transcription factor-related Pfam domains.** Transcription factor-related Pfam domains were identified using Pfam domain assignment. Expression values as represented by fragments per kilobase pair of exon model per million fragments mapped (FPKM) were calculated using Cufflinks [58].
(XLS)
