# Supplementary figures and images for: Expression Profiling of Cucumis sativus in Response to Infection by Pseudoperonospora cubensis
Source: PLoS One. 2012 Apr 24;7(4):e34954. doi: 10.1371/journal.pone.0034954 (PMC3335828; doi:10.1371/journal.pone.0034954)

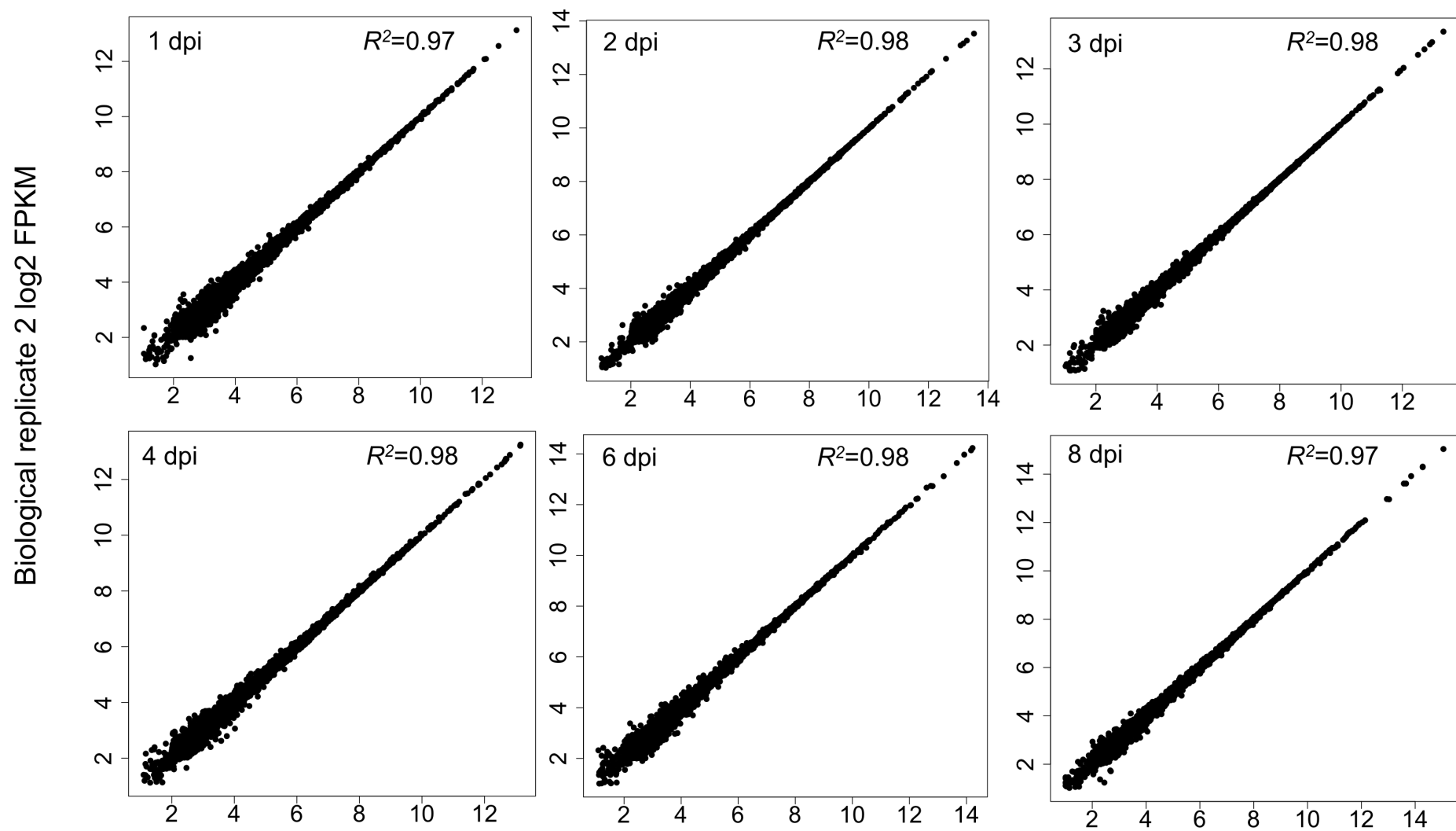

Biological replicate 1 log2 FPKM

Figure S1, Adhikari et al.

Supplement: Figure S1 — Concordance of expression values in two biological replicates of Cucumis sativus during infection by Pseudoperonospora cubensis . Reads from different time points were mapped to the C. sativus genome using Bowtie version 0.12.5 [57] and TopHat version 1.2.0 [56]. Fragments per kilobase pair of exon model per million fragments mapped (FPKM) values were calculated using Cufflinks version 0.9.3 [58] and the C. sativus genome annotations. For each time point, log2 transformed FPKM values of equal number of genes from both replicates are plotted. R2, correlation coefficient; dpi, days post-inoculation. (PDF) [file pone.0034954.s001.pdf]

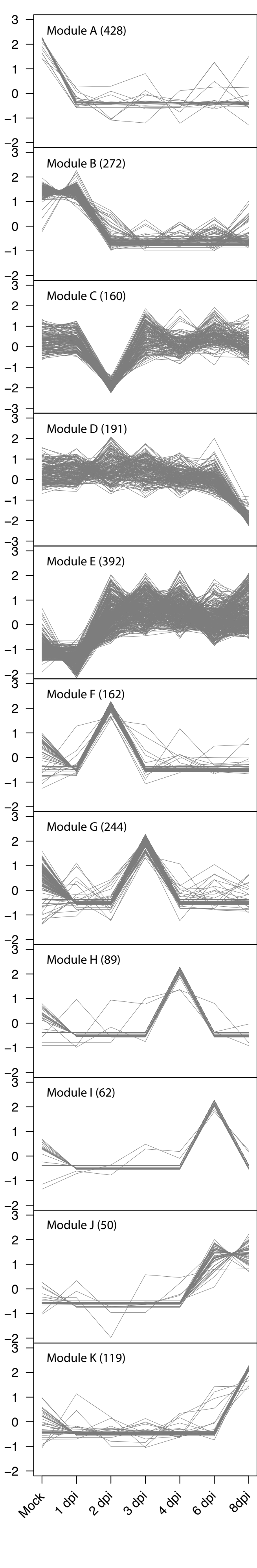

Supplement: Figure S2 — Trend plots for all 11 modules. All 11 modules generated using WGCNA are shown (Modules A through K). The number of genes in each module is shown in parentheses. (PDF) [file pone.0034954.s002.pdf]
